# Deficit Irrigation Effects on Cotton Growth Cycle and Preliminary Optimization of Irrigation Strategies in Arid Environment

**DOI:** 10.3390/plants13101403

**Published:** 2024-05-17

**Authors:** Meiwei Lin, Lei Wang, Gaoqiang Lv, Chen Gao, Yuhao Zhao, Xin Li, Liang He, Weihong Sun

**Affiliations:** 1 School of Agricultural Engineering, Jiangsu University, Zhenjiang 212013, China; 2212216002@stmail.ujs.edu.cn (M.L.); gqlv@ujs.edu.cn (G.L.); 2212216007@stmail.ujs.edu.cn (C.G.); 2212316004@stmail.ujs.edu.cn (Y.Z.); 2212316010@stmail.ujs.edu.cn (X.L.); 2 School of Computer Science and Technology, Xinjiang University, Urumqi 830017, China; 107552201335@stu.xju.edu.cn; 3 Department of Electronic Engineering, and Beijing National Research Center for Information Science and Technology, Tsinghua University, Beijing 100084, China

**Keywords:** cotton, deficit irrigation, arid environment, low-soil quality improvement, sustainable agriculture, DSSAT

## Abstract

With the changing global climate, drought stress will pose a considerable challenge to the sustainable development of agriculture in arid regions. The objective of this study was to explore the resistance and water demand of cotton plants to water stress during the flowering and boll setting stage. The experimental plot was in Huaxing Farm of Changji city. The plots were irrigated, respectively, at 100% (as the control), 90%, 85% and 80% of the general irrigation amount in the local area. The relationship between the various measured indexes and final yield under different deficit irrigation (DI) treatments was studied. The results showed that deficit irrigation impacted the growth and development processes of cotton during the flowering and boll setting stage. There was a high negative correlation (*R*^2^ > 0.95) between the maximum leaf area index and yield. Similarly, there was a high correlation between malondialdehyde content and yield. Meanwhile, 90% of the local cotton irrigation contributed to water saving and even increasing cotton yield. Furthermore, based on the results, the study made an initial optimization to the local irrigation scheme by utilizing the DSSAT model. It was found that changing the irrigation interval to 12 days during the stage could further enhance cotton yield and conserve resources.

## 1. Introduction

Plants face various environmental stresses during their entire lifecycle [1]. Due to the rise of global temperatures and the severity of drought, plants are more vulnerable to abiotic stresses in arid regions [2,3]. Drought is becoming a major environmental constraint to the sustainability of agriculture on a global scale [4,5].

Cotton (*Gossypium hirsutum* L.) is an important economic crop worldwide [6]. Among all abiotic stresses, drought presents the most formidable threat to the entire growth and development process of cotton [3]. Although cotton is a warmth-loving crop, the flowering and boll setting stage of cotton (from July to October in China) frequently leads to the reduction in yield, which is particularly pronounced in arid regions [3,7,8,9]. Therefore, studying the impact of saving irrigation on cotton yield and quality formation during these development stages can provide a basis for exploring the high-quality cultivation of cotton.

China is one of the top ten cotton-producing countries globally, with its largest cotton-producing region being the Xinjiang Uygur Autonomous Region [2,10,11]. Xinjiang is located in the northwest inland of China and is situated in an arid region with a shortage of water resources and low agricultural-water use-efficiency [12,13]. In the face of these issues, how to reasonably utilize the water resources and increase agricultural ecological efficiency will be a major breakthrough in the sustainability of agriculture in Xinjiang [14,15]. Furthermore, due to Xinjiang’s expansive territory, the uneven distribution of water resources has led to varying pressures across its cotton planting regions [16]. Clearly, the implementation of water-saving irrigation is the fundamental pathway for the development of cotton cultivation in Xinjiang [17]. Therefore, investigating water requirements during the different stages of crop life and then providing a precise irrigation strategy is important. It will contribute to achieving high production and ecological efficiency in cotton fields in Xinjiang.

Regulated Deficit Irrigation (RDI) is considered to be a key practice in the utilization of limited water resources [18]. This approach aims to enhance crop water use efficiency and yields by relying on the plant’s self-regulation and supplementary effects [19,20]. A number of studies have demonstrated that the use of deficit irrigation could offer significant advantages in the context of limited water supplies [21,22]. Optimizing the irrigation volume has been shown to not only increase yield but also maintain cotton quality [22,23,24]. By employing this approach, it also can reduce the dependency on fertilizers and pesticides, resulting in decreasing environmental pollution [19,25]. Nonetheless, to some extent, improper RDI may also result in negative environmental consequences. Excessive RDI could hinder crop growth and adversely affect agricultural production. Moreover, water stress can lead to the onset and propagation of crop pests and diseases [26]. Therefore, it is essential to adjust the degree of RDI based on the physiological characteristics of crops and the environmental conditions at that time.

Furthermore, combining suitable irrigation decision support systems will be conducive to promote the implementation of water-saving technologies. DSSAT (Decision Support System for Agrotechnology Transfer) provides a simulation platform for evaluating the impact of different management strategies on crop growth and yield [27,28,29,30]. It also holds a significant position in irrigation decision support systems. It can simulate crop growth and irrigation water requirements under different irrigation strategies [29,31]. As evidenced by empirical studies, the DSSAT model is capable of not only simulating the impact of stress on cotton growth but also serving as a reference for cotton planting and irrigation management under diverse climatic conditions [32,33]. Therefore, DSSAT can assist agricultural producers in optimizing irrigation plans and making more scientific and sustainable irrigation decisions [29,34].

In summary, in the context of the high use of water and fertilizer in Xinjiang, it is essential to conserve water and increase the productivity of the land. Meanwhile, the study of deficit irrigation will contribute to sustainable agricultural development. So, the effects of different RDI treatments on cotton growth, yield, and physiological characteristics during the flowering and boll setting stage were studied. The findings could provide a theoretical basis for exploring water demand regulation to maintain the yield under water stress. Furthermore, the local irrigation schemes would be preliminarily optimized to adapt to ever-changing climatic conditions by using the DSSAT model.

## 2. Materials and Methods

### 2.1. Experimental Site and Plant Material

**Figure 1:**
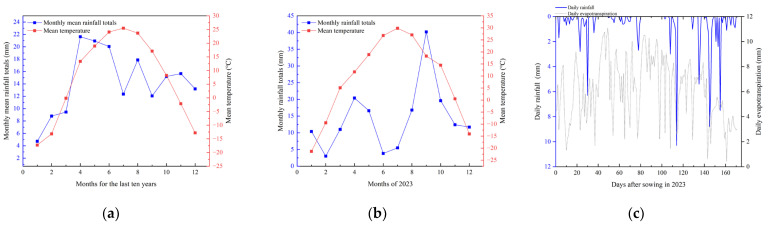
The meteorological data for the last ten years (**a**) and for the research year 2023 (**b**,**c**).

The field trial was conducted on Huaxing Farm (44.22° N, 87.29° E, 31 m altitude) in Changji City of the Xinjiang Uygur Autonomous Region. The field experiment data were collected from April to October in 2023. The average annual rainfall in the experimental site was 190 mm. The effective accumulated temperature was 3450 °C, and there were 160 to 190 frost-free days during the whole year. In addition, the soil properties and the meteorological data in the testing field were shown in Table 1 and Figure 1, respectively.

**Figure 2:**
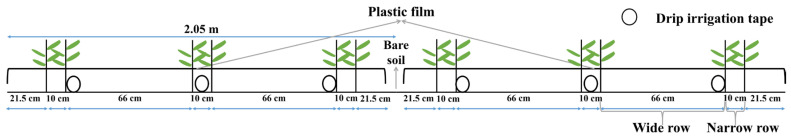
Layout of drip irrigation for cotton.

The plant material was ZhongMian 113 (*Gossypium hirsutum* L.) which was one of the local recommended cotton varieties. It had the characteristics of extra early maturing and high fiber content. The cotton was sown on 23 April 2023 and harvested on 15 October 2023. Its cropping pattern was under-mulch drip irrigation as shown in Figure 2. Three tubes were laid out in six cotton planting rows with one mulch film (66 cm + 10 cm), and a wide–narrow row was present. The plants were divided into wide (66 cm) and narrow (10 cm) rows. The plant spacing was 8 to 10 cm and the sowing density was 210,000 plants per hectare. 

### 2.2. Experimental Design

The amount of general region irrigation in the whole growth period of cotton was 5400 m^3^/ha. Based on this value, four water gradients were set up, including W100 as the control (full irrigation without water stress, 5400 m^3^/ha), W90 as mild deficit treatment (10% less than the control, 4860 m^3^/ha), W85 as moderate deficit treatment (15% less than the control, 4590 m^3^/ha) and W80 as severe deficit treatment (20% less than the control, 4320 m^3^/ha). Its irrigation frequency and fertilizer application were consistent with the cotton fields on the farm. During our experiment, considering that the rainfall from May to August of 2023 was less than the average rainfall during the same period in previous years (Figure 1), the irrigation time interval was about 7 days during the flowering and boll setting stage of cotton. This was to ensure that the water needs of the control group were met. Table 2 showed the total irrigation amount in different growth periods of cotton plants. There were multiple irrigation operations during each growth period. Every irrigation time or amount was slightly different per growth period based on soil moisture content, crop status and climatic conditions.

**Figure 3:**
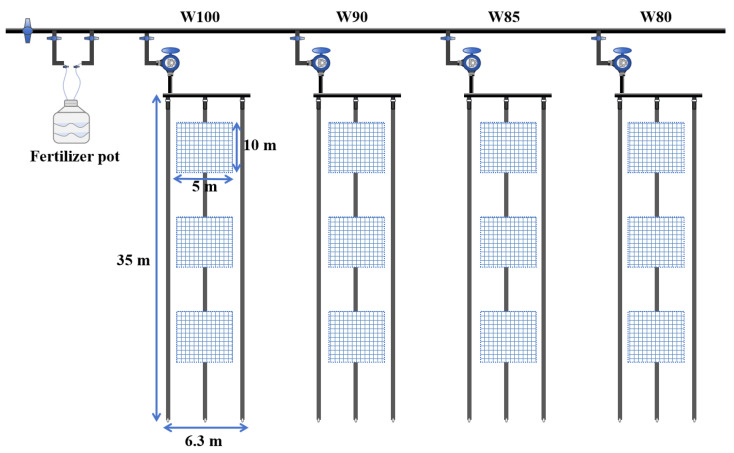
Schematic diagram of the field experiment.

All of the experimental plots were 35 m in length and 6.3 m in width, as shown in Figure 3. There were three sampling areas in each experimental plot as a repetition, which were located in the front, middle, and rear sections of each plot, respectively. Each sampling area was 10 m in length and 5 m in width. During the flowering and boll setting stage of cotton, the plants were measured every seven days in each plot.

### 2.3. Determination of Growth and Development Traits of Cotton Plants during Water Treatment Phase

Nine plants with a uniform growth were selected, marked with tags, and subjected to regular surveys. Measurements included plant height (from the cotyledon node to the top of the main stem), stem diameter (width at a point 1 cm above the cotyledon node), and leaf area index (perforation method). 

The leaf area index was measured according to perforation method. Three plants were taken from each plot every seven days, and the leaves of all the plants were removed and punched with a test punch (10 cm in diameter). Then, the punched and unpunched leaves were bagged separately and placed in an oven for drying and weighing. Finally, the leaf area index was calculated using the following Formulas (1) and (2).

Leaf area = number of punched leaves × area of a single hole/dry weight of perforated leaves × (dry weight of punched leaves + dry weight of unpunched leaves).(1)

Leaf area index = total leaf area/occupied area(2)

### 2.4. Measurement of Cotton Boll Formation Characteristics during the Water Treatment Phase

After cotton flowering, the first fruiting branch node, the number of fruiting branches per plant, the number of flowers, buds and bolls per plant were measured for tagged cotton plants. The growth and shedding of buds, flowers and bolls were counted, and the boll-setting rate and shedding rate were calculated accordingly.

### 2.5. Determination of Cotton Biomass during the Water Treatment Phase

Nine representative cotton plants were randomly selected. The plants were separated into stems, leaves, and reproductive organs (buds, flowers and bolls). The samples were killed out at 105 °C for 30 min, then dried at 75 °C until a constant weight was achieved. The dry matter weight of each organ were recorded.

### 2.6. Determination of Stress Resistance Indicators in Cotton Functional Leaves during the Water Treatment Phase

Three days after the third time irrigation of the stage (fourteen days after the cotton flowering), the functional leaves of plants were randomly taken. The parameters of the leaves were measured according the methods of Gao et al. [35], including superoxide dismutase (SOD) activity, peroxidase (POD) content, malondialdehyde (MDA) content, and soluble protein (SP) content.

### 2.7. Calculation of Cotton Yield and Yield Components under Water Treatment

Within each treatment test plot, a 6.67 square meter area with uniform growth and plant density randomly selected, and the total boll count (including opened and unopened bolls) and the number of unopened cotton bolls were counted. After drying, the harvested cotton were weighed and calculated using the following Formulas (3) and (4).

Seed cotton yield = Plant density × Boll count per plant × Weight of a single boll(3)

Lint cotton yield = Seed cotton yield × Lint percentage(4)

### 2.8. Calculation of Field Irrigation Water Use Efficiency

The irrigation water use efficiency in the cotton field was calculated using the following Formula (5). IWUE represented the irrigation water use efficiency as a unit of kg·m^−3^; Y was the unginned cotton yield of the test field as a unit of kg·hm^−2^, and I was the total irrigation water applied during the entire growth period of tested cotton field, as a unit of m^3^·hm^−2^.

IWUE = Y/I(5)

### 2.9. Data Processing

The experimental data were presented as mean ± standard error. A single-factor analysis of variance was employed to assess the significance of differences in a specific measurement index among different treatments. Multiple comparisons were conducted by using the LSD test. The statistical analysis was performed by using SPSS 25.0 software. The data were organized by using Microsoft Excel 2021 before generating plots with Origin 2021.

### 2.10. Optimization of Irrigation Strategies during the Flowering and Boll Setting Stage of Cotton

Through importing meteorological, soil, and crop data, the parameters of the cotton module in the DSSAT model were adjusted. The data used in parameter adjustment were obtained as follows: the weather data were obtained from a weather station near the test site (43.9° N, 87.4° E), soil data were prepared from a weather station near the test site at “NASA GES DISC” (https://ldas.gsfc.nasa.gov/gldas/, accessed on 7 January 2023) and the scattered historical climatic data of Changji were calibrated to derive the crop data. Then, using the calibrated DSSAT model, the different irrigation treatments during the cotton flowering and boll setting stage were inputted to simulate the final yield. Subsequently, according to the simulation results, it would provide an optimization strategy of the irrigation scheme in local cotton fields.

## 3. Results

### 3.1. The Effect of Different Irrigation Levels on the Growth and Development of Cotton during the Flowering and Boll Setting Stage

#### 3.1.1. The Plant Height and Stem Diameter of Cotton under Different Irrigation Treatments

**Figure 4:**
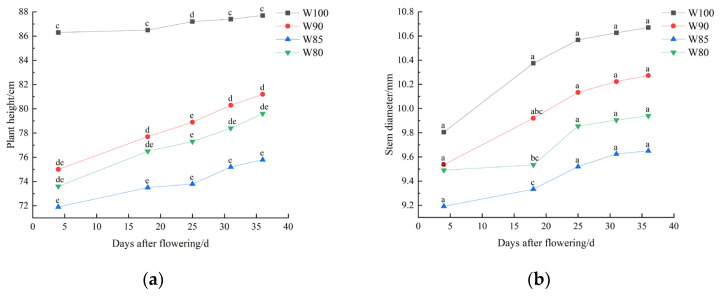
Changes in cotton plant height (**a**) and stem diameter (**b**) under different irrigation treatments. Note: different lowercase letters indicate significant differences within different treatments (*p* < 0.05).

As shown in Figure 4, the growth amplitude of the cotton plant height was small, and then gradually tended to stabilize. The rapid growth of the stem diameter occurred between the 20th and 25th days after flowering.

Plant height significantly decreased with decreasing irrigation levels, compared to the control group (Figure 4a). However, no statistically significant difference in plant height was observed among deficit irrigation treatments. Similarly, the cotton stem diameter decreased with the reduction of irrigation levels (Figure 4b), but there were also no significant differences. Additionally, a cubic polynomial could effectively simulate the changes in cotton plant height and stem diameter with the growth days under different irrigation treatments (*R*^2^ > 0.97) by fitting data (Table 3). 

#### 3.1.2. The Leaf Area Index of Cotton under Different Irrigation Treatments

**Figure 5:**
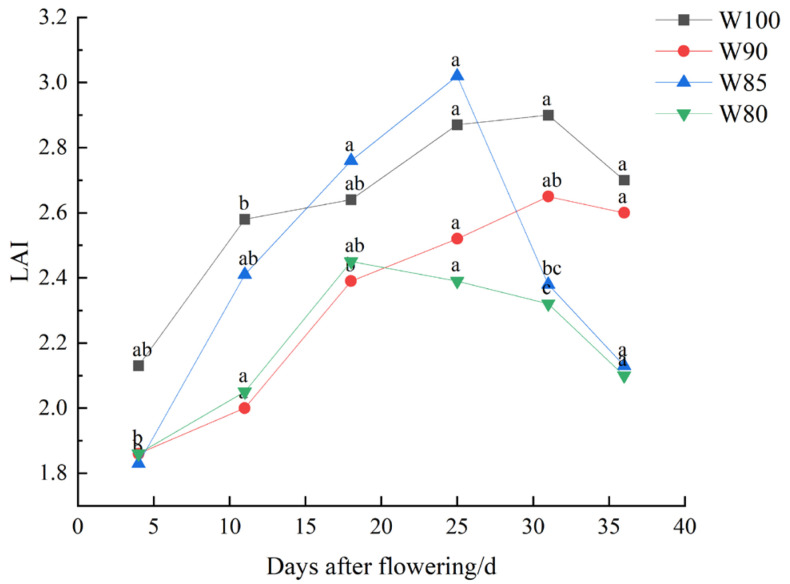
Changes in cotton leaf area index under different irrigation treatments. Note: different lowercase letters indicate significant differences within different treatments (*p* < 0.05).

As shown in Figure 5, the leaf area index showed a total trend of initial increase followed by a later decrease. It demonstrated diverse fluctuations under reduced irrigation levels. While an overall decrease was observed, there was nothing statistically significant. However, the deficit irrigation primarily influenced the time point when the leaf area index reached its maximum value. As irrigation levels decreased, the shedding time of cotton leaves advanced. Additionally, by fitting data, a cubic polynomial could effectively simulate the changes in cotton leaf area index with the growth days under different irrigation treatments (*R*^2^ > 0.92) (Table 3). 

### 3.2. The Effect of Different Irrigation Levels on the Boll-Setting Characteristics of Cotton during the Flowering and Boll Setting Stage

**Figure 6:**
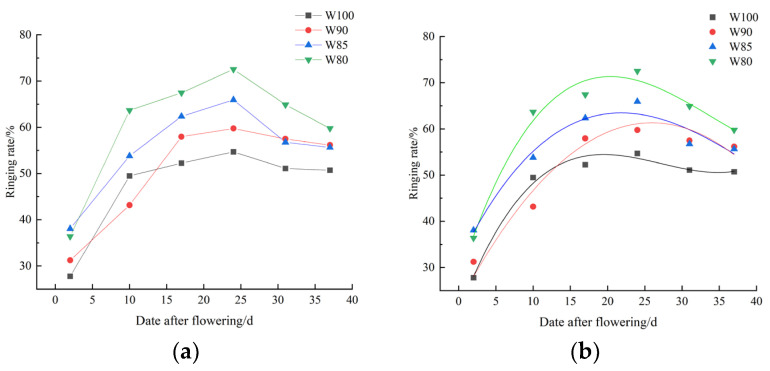
Changes in cotton ringing (**a**) under different irrigation treatments, along with its fitted curve (**b**).

**Figure 7:**
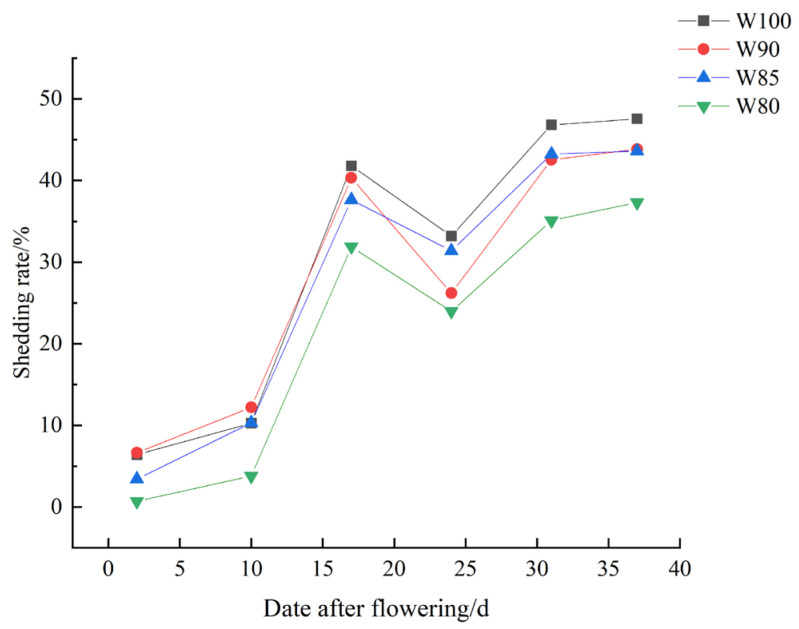
Changes in cotton shedding under different irrigation treatments.

As shown in Table 4, Figure 6 and Figure 7, the number of fruiting branches and bolls gradually increased with the increase in growth days. Concurrently, the number of buds and young bolls decreased gradually. The ringing rate exhibited an initial increase and then decreased. The shedding rate showed a trend of rapid increase, and then followed by changes in both decrease and increase.

#### 3.2.1. The Ringing Rate of Cotton Plants under Different Irrigation Treatments

As shown in Figure 6, the ringing rate showed a trend of initially increasing and then decreasing, with a significant early stage increase and a smaller decrease in the later stage. Its peak value was primarily around the 25th day after flowering. 

As the irrigation levels decreased, the peak value of the ringing rate gradually increased (W80 > W85 > W90 > W100). Moreover, through fitting the data, Table 3 showed that a third-degree polynomial could simulate the changes well in the ringing rate of cotton plants under different irrigation treatments (*R*^2^ > 0.93).

#### 3.2.2. The Shedding Rate of Cotton Plants under Different Irrigation Treatments

As shown in Figure 7, the shedding rate exhibited a pattern of rapid increase followed by a decrease, then a slow increase, with its lowest value also primarily around the 25th day after flowering. Compared to the control, it gradually decreased with the reduction in irrigation levels, and there was a greater reduction in shedding rate with the more water deficient treatments (Figure 7).

### 3.3. The Effect of Different Irrigation Levels on the Biomass Accumulation and Distribution of Cotton during the Flowering and Boll Setting Stage

**Figure 8:**
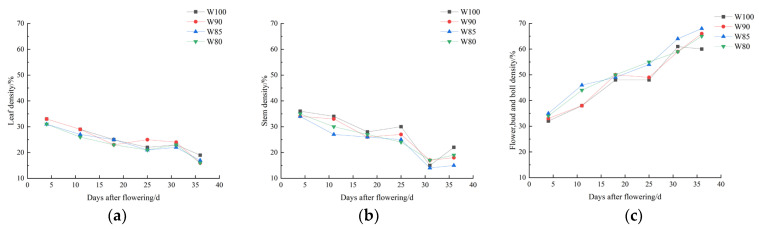
The changes in the biomass proportions of various organs of cotton under different irrigation treatments. (**a**) The biomass proportions of leaves; (**b**) the biomass proportions of stems; (**c**) the biomass proportions of flowers, buds and bolls.

As shown in Figure 8, the proportion of above-ground biomass accumulation in cotton exhibited diverse changes. With the increase in growth days, the proportion of flowers, buds and bolls showed an increasing trend (Figure 8c), while the proportions of leaves and stems showed a decreasing trend (Figure 8a,b). In comparison to the control, the decrease in irrigation levels led to a reduction in the proportions of leaves and stems, accompanied by an increase in the proportions of flowers, buds and bolls.

### 3.4. The Effect of Different Irrigation Treatments on the Stress Resistance within Functional Leaves of Cotton during the Flowering and Boll Setting Stage

#### 3.4.1. The Changes in the Activity of Endogenous Protective Enzyme Systems within Functional Leaves of Cotton under Different Irrigation Treatments

The strength of a plant’s ability to resist external stress is correlated with the level of the cellular antioxidant system. This system consists of both enzymatic antioxidant enzymes and non-enzymatic antioxidant substances. Among them, superoxide dismutase (SOD) and peroxidase (POD) were crucial enzymes in the enzymatic antioxidative system. 

**Figure 9:**
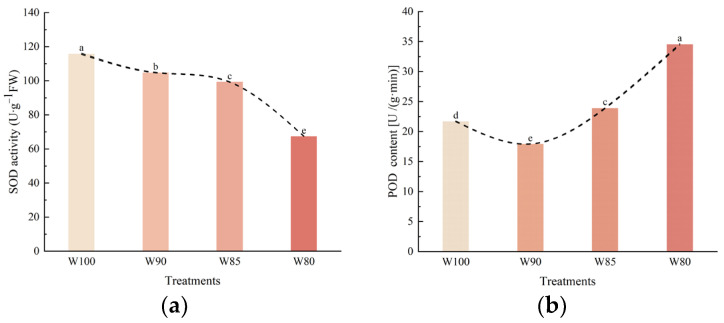
The effect of different irrigation treatments on the endogenous protective enzyme system within functional leaves of cotton. (**a**) The activity of superoxide dismutase; (**b**) the content of peroxidase. Note: different lowercase letters indicate significant differences within different treatments (*p* < 0.05).

Compared to the control, the SOD activity of the functional leaves of cotton slightly remained stable in the mild water deficit, but showed a decreasing trend in the other water deficits (Figure 9a). Figure 9b showed that the POD content exhibited a trend of initial decrease followed by an increase with the reduction in irrigation levels.

#### 3.4.2. The Changes in Soluble Protein and Malondialdehyde Content in Functional Leaves of Cotton under Different Irrigation Treatments

**Figure 10:**
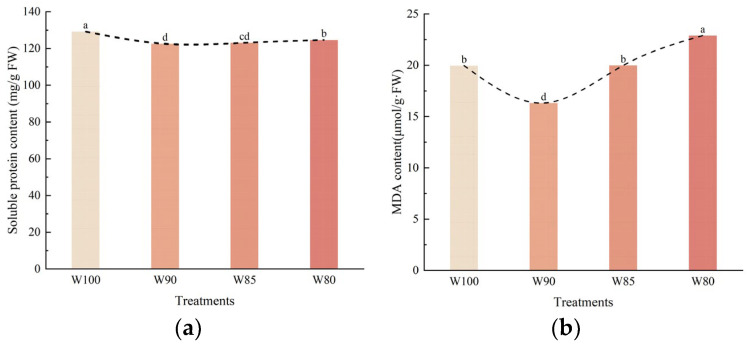
The effect of different irrigation treatments on the soluble protein (**a**) and malondialdehyde content (**b**) within functional leaves of cotton. Note: different lowercase letters indicate significant differences within different treatments (*p* < 0.05).

Soluble protein serves as a critical osmoregulation substance, serving as a vital indicator of plant drought resistance. Its content is intricately linked to the cellular water retention capacity, providing protection to vital cell substances and biofilms. As shown in Figure 10a, different irrigation levels significantly influenced the soluble protein content in functional leaves. In comparison to the control, the soluble protein content in leaves decreased with the reduction in irrigation levels. However, as the degree of irrigation reduction intensified, there was a subsequent recovery in the soluble protein content in cotton plants.

Malondialdehyde (MDA) is a byproduct of membrane lipid peroxidation in plants under adverse conditions. Its concentration serves as an indicator of the extent of damage to the plant cell membrane system under environmental stress. As shown in Figure 10b, MDA content in functional leaves significantly increased under severe stress. The MDA content of W90 was lower than other treatments.

### 3.5. The Effect of Different Irrigation Treatments on the Yield Formation of Cotton

#### 3.5.1. Cotton Yield and Its Constituent Factors under Different Irrigation Treatments

As shown in Table 5, the yield exhibited an initial increase followed by a decreased trend with the reduction in irrigation levels. Regarding yield components, the number of bolls exhibited the most significant variation. Compared with the control, the number of bolls decreased, while both the single boll weight and lint percentage increased, with varying degrees of changes.

Clearly, the increase in yield for the W90 treatment was primarily due to a significant increase in single boll weight, while the decrease in yield for the W85 and W80 treatments was mainly attributed to a substantial reduction in the number of bolls. It must be noted that the results were preliminary of the one-year yields and the field tests.

#### 3.5.2. The Relationship between Cotton Yield and Irrigation Water Use Efficiency

**Figure 11:**
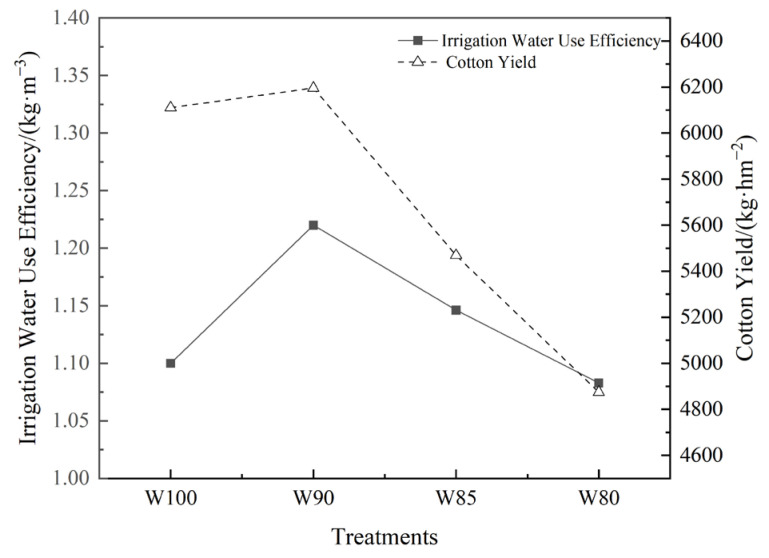
The mutual relationship between irrigation water use efficiency and yield in cotton plants.

Through the analysis of cotton yield and irrigation water use efficiency under different irrigation treatments, the results indicated that cotton yield and irrigation water use efficiency exhibited a similar trend (Figure 11). With the decrease in irrigation levels, the cotton yield and irrigation water use efficiency of each treatment was W90 > W100 > W85 > W80. The W90 treatment showed the highest irrigation water use efficiency and yield, reaching 1.22 kg/m^3^ and 6196 kg/hm^2^, respectively. It needed to be noted that the results were preliminary of the cotton yield and water use efficiency on one-year field tests.

### 3.6. The Correlation Analysis of Yield and Measured Indicators during the Flowering and Boll Setting Stage of Cotton under Different Irrigation Treatments

There was a certain correlation between cotton yield and yield components, as well as various measured indicators of cotton (Table 6). In terms of growth and development, maximum LAI was significantly negatively correlated with yield. Additionally, MDA content and POD content were also significantly correlated with yield in terms of stress resistance. Furthermore, the correlation rankings for each type of indicator with yield were presented in Table 6.

### 3.7. Optimizing Irrigation Schemes for Cotton during the Flowering and Boll Setting Stage

For the considered appropriate irrigation strategy (mild deficit water treatment W90), this study showed the further allocation of irrigation frequency and volume during the flowering and boll setting stage of cotton in Table 7.

**Figure 12:**
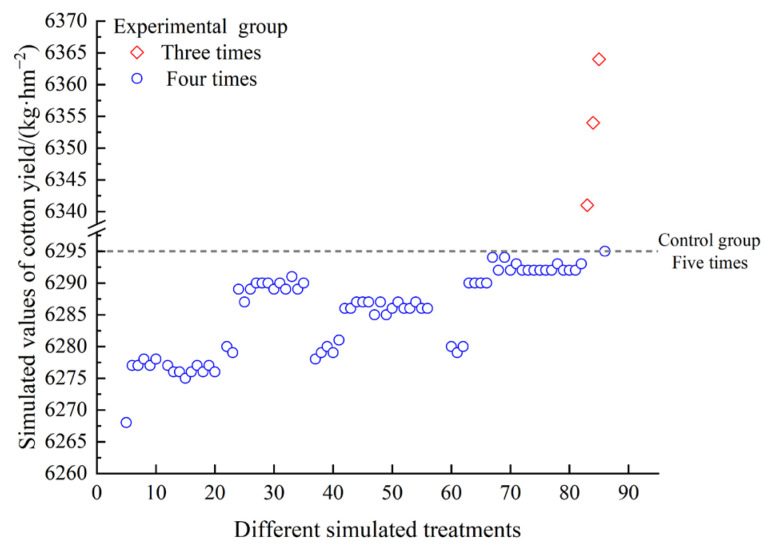
Simulation of cotton yield under different water allocations.

Based on the previous research of Wang et al. [36], the parameters of the cotton model in DSSAT were adjusted. Then, the DSSAT was used to simulate the final yield of cotton after the different water treatments. The results indicated that if the irrigation frequency was three times, with an irrigation interval of 12 days, the yield would be superior to the control group. However, when the irrigation frequency was four times, with an irrigation interval of 9 days, the yield was less than or close to that of the control group (five times) (Figure 12).

## 4. Discussion

**Figure 13:**
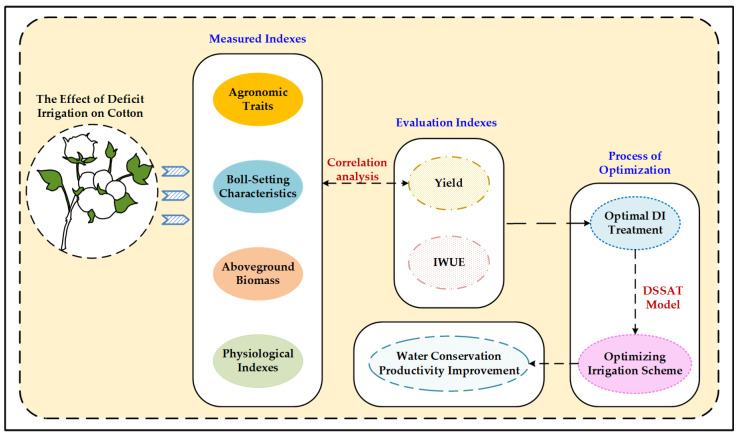
The technical route of deficit irrigation.

The technical route of this study was shown in Figure 13. The aim was to make adjustments to water-saving irrigation in response to general region norms of cotton irrigation, and to study the effect of deficit irrigation on cotton by measuring the morphological and internal characteristics of plants. The effectiveness of deficit irrigation was evaluated from two main aspects: yield and water use efficiency, in order to select the optimal deficit irrigation treatment. In addition, irrigation scheme was further optimized by using the DSSAT model to achieve the goals of water conservation and productivity improvement, while promoting sustainable agricultural development. Furthermore, it was important to note that the results were preliminary and the field tests with irrigated plants would continue to evaluate cotton yield and irrigate indicators in subsequent growing seasons.

Although the effects of a regulated deficit irrigation on crops has been studied, the results were limited [18,19,37]. There was still a need to improve the applicability of RDI in arid areas. In addition, most of the existing studies were limited to obtaining the optimal level of RDI in the experiment [38]. Few studies had been undertaken to combine RDI with an irrigation decision support system. The results of this study would help to improve the implementation and application of this water-saving technology. 

The external features of a plant is one of the direct phenotypes they display in response to water stress [39]. The overall growth trend of cotton shown in Figure 4 and Figure 5 were similar with the results of Li et al. [40]. Cotton plants showed a decrease in plant height, stem diameter and leaf area index under DI treatments. These findings were consistent with the research results on the impact of water deficit stress on cotton growth [41,42]. However, it was notable that water stress primarily influenced the plant height of cotton and the maximum value of leaf area index (Figure 4a and Figure 5). 

Furthermore, the ringing rate increased and the shedding rate decreased under deficit irrigation (Figure 6 and Figure 7). In other words, cotton plants tended to begin flower shedding early when entering the period of boll setting, in order to utilize limited substances and energy. Similarly, the biomass accumulation of flowers, buds and bolls showed a more significant increasing trend than those of the leaves and stems (Figure 8). It might be a morphological phenomenon of plant priority selection under water stress [40]. Cotton mainly coped with the water stress by advancing reproductive growth, thereby reducing the losses. 

As mentioned above, it was clear that DI affected the growth and development processes of cotton. Studies had shown that water stress inhibited the vegetative growth of cotton and stimulated the drought resistant mechanisms, consequently beginning boll setting earlier [38,43]. In our study, mild DI did not impose severe stress on the growth of cotton and was more favorable for the reproductive growth. This contributed to achieving water-saving characteristics and promoting an increased yield. Moreover, a cubic polynomial could well simulate the changes in cotton plant height (*R*^2^ > 0.98), stem thickness (*R*^2^ > 0.97), leaf area index (*R*^2^ > 0.92) and ringing rate (*R*^2^ > 0.93). Therefore, it could be used to reflect the growth of cotton during the flowering and boll setting period. 

Several physiological indexes within functional leaves can reflect the resistance mechanisms of plants. The results showed that DI treatments significantly affected osmotic regulation substances, the endogenous protective enzyme, and cell membrane permeability in the leaves (Figure 9 and Figure 10).

Under water stress, the content of soluble protein was generally reduced. It might be because that water stress inhibited the protein synthesis pathway of the cell in leaves [2,44]. However, with the degree of DI deepening, the content of soluble protein was increased slightly (Figure 9a). With the level of stress rising, the plant’s stress response system was triggered, and the expression of stress resistant genes increased. Therefore, a corresponding rise of soluble protein content was translated by some stress resistant genes [45]. In addition, water stress attacks the cell membrane structure and leads to an increase in MDA [46]. The results indicated that mild stress activated the plant’s defense system, and the generation of reactive oxygen species was controlled, thereby the generation of MDA was reduced [47,48,49]. 

Similarly, the excess reactive oxygen species attacked the antioxidant enzyme system under water stress, resulting in SOD activity decreasing. Additionally, mild drought stress and the accumulation of reactive oxygen species further induced POD expression. Then, the excessive reactive oxygen species within the cells were quickly cleaned up, and it alleviated the extent of oxidative damage in leaves. On the whole, it was observed that a mild DI contributed to the better regulation of endogenous protective enzyme systems, stabilizing cell membrane structure and function in cotton plants (Figure 9 and Figure 10).

DI may optimize water management and usage [50]. In order to understand the effect of DI on actual production and water resource utilization, the final yield and IWUE were measured. The appropriate DI mainly influenced the yield by increasing the single boll weight (Table 5). The most significant factor of yield losses was the reduction in bolls, which was consistent with the findings of other studies [49,51]. However, the contribution rates of these three factors to yield were not fixed or absolute. In addition, W90 treatments not only improved the irrigation water use efficiency but also increased the yield (Figure 11). W90 treatments contributed to achieving the goal of water conservation and the maintenance of yield. 

Meanwhile, for the sake of exploring water demand regulation to maintain cotton yield under drought, the relationship between the measured indexes and the yield were analyzed. Table 6 indicated that the maximum leaf area index was significantly negatively correlated with the yield, and the MDA content was negatively correlated with the yield too. Additionally, the maximum ringing rate, maximum shedding rate and number of bolls showed relatively high correlations with the yield (>0.85), respectively. It could be a preferred irrigation scheme for cotton plants in arid areas.

Furthermore, it is crucial to adopt appropriate irrigation strategies and optimize the yield with respect to available water quantity for production under conditions of water scarcity [37,52]. To further explore the possibility of reducing irrigation costs and to promote sustainable agricultural development, the irrigation times and amount during the flowering and boll setting stage were studied by using the DSSAT model. According to the optimal DI treatment W90, it was conducive to increasing the yield and conserving resources, when the irrigation frequency was three times and the irrigation interval was 12 days (Figure 12).

## 5. Conclusions

In the context of the high use of water and fertilizer in Changji and its surrounding areas of Xinjiang, the impact of deficit irrigation treatments on the flowering and boll setting stage of cotton were studied in the aspects of growth, physiological characteristics and yield. The results indicated that crop height, stem diameter and leaf area index were significantly reduced under deficit irrigation. Deficit irrigation significantly affected osmotic regulation substances, the endogenous protective enzyme and cell membrane permeability in the leaves. The preliminary results of this year’s field tests showed that 90% of the local cotton irrigation (mild deficit treatment) contributed to water saving and even increasing cotton yield. Furthermore, based on the DSSAT model, it was shown that when the irrigation interval is 12 days, it could further increase the cotton yield in the area and water resources. The study of the verification irrigation volume and the prediction irrigation intervals could provide a scientific basis for the study of the water-saving irrigation methods of cotton planting in subsequent growing seasons.

## Figures and Tables

**Table 1:** Soil physical and chemical properties (0–100 cm).

Soil Layer (cm)	Clay (%)	Silt (%)	Sand (%)	Bulk Density (g/cm^3^)	Field Moisture (%)	Soil Porosity	EC (ms/cm)
0–10	2.91	16.89	80.20	1.062	0.186	0.599	1.53
10–20	3.08	17.86	79.06	1.251	0.223	0.528	1.38
20–30	2.73	16.99	80.28	1.300	0.216	0.509	1.12
30–40	3.07	19.44	77.49	1.252	0.219	0.527	1.72
40–60	3.02	18.91	78.07	1.252	0.206	0.528	1.40
60–80	2.66	17.22	80.12	1.266	0.236	0.522	1.51
80–100	2.68	17.78	79.54	1.245	0.221	0.530	1.30

**Table 2:** Irrigation amounts during whole growth period of cotton under different treatments.

Treatments	Irrigation Amount/(m^3^·ha^−1^)					Total Irrigation Amount
	Sprouting	Seedling	Bud Emergence	Flowering and Boll Setting	Boll Opening	
W100	450	525	1575	2550	300	5400
W90	450	465	1410	2265	270	4860
W85	450	435	1320	2130	255	4590
W80	450	405	1245	1980	240	4320

**Table 3:** The fitting equations for the changes in cotton plant height, stem diameter, leaf area index, and ringing rate under different irrigation treatments.

Indicators	Treatments	Fitting Equation	*R* ^2^
Plant height	W100	y = 86.67 − 0.1283x + 0.0092x^2^ − 0.0001x^3^	0.9825
	W90	y = 74.24 + 0.1918x − 0.0003x^2^ + 0.00001x^3^	0.999
	W85	y = 71.29 + 0.1744x − 0.0052x^2^ + 0.00011x^3^	0.9804
	W80	y = 72.04 + 0.4513x − 0.0160x^2^ + 0.00026x^3^	0.9997
Stem diameter	W100	y = 9.59 + 0.0578x − 0.0007x^2^ − 0.0000009x^3^	0.9991
	W90	y = 9.47 + 0.0123x + 0.0012x^2^ − 0.00002x^3^	0.999
	W85	y = 9.29 − 0.0351x + 0.0029x^2^ − 0.00005x^3^	0.9997
	W80	y = 9.74 − 0.0841x + 0.0057x^2^ − 0.00009x^3^	0.9738
LAI	W100	y = 1.94 + 0.056x − 0.00036x^2^ − 0.00001x^3^	0.9433
	W90	y = 1.81 + 0.014x − 0.0025x^2^ − 0.00005x^3^	0.9841
	W85	y = 1.37 + 0.1167x − 0.0014x^2^ − 0.00004x^3^	0.9222
	W80	y = 1.68 + 0.0369x − 0.0008x^2^ − 0.00004x^3^	0.9341
Ringing rate	W100	y = 0.20 + 0.0434x − 0.0017x^2^ + 0.00002x^3^	0.9873
	W90	y = 0.26 + 0.0243x − 0.0004x^2^ − 0.000001x^3^	0.9665
	W85	y = 0.31 + 0.0331x − 0.0010x^2^ + 0.00001x^3^	0.9572
	W80	y = 0.27 + 0.0503x − 0.0018x^2^ + 0.00002x^3^	0.9341

**Table 4:** The changes in the boll-setting characteristics under different irrigation treatments.

Treatments	Dates after Flowering	Fruiting Branches	Buds	Flowers	Young Bolls	Bolls
W100	2	9.3	6.6	3.1	2.2	2.1
	10	10.5	2.3	3.4	1.4	5.4
	17	10.7	0.9	0.8	0.5	6.5
	24	10.9	0.3	0.6	0.2	6.3
	31	11.0	0.1	0.2	0.1	6.2
	37	11.6	0.2	0.0	0.2	6.4
W90	2	10.4	6.3	4.0	1.1	3.8
	10	10.8	1.8	5.1	0.8	5.7
	17	10.9	0.4	1.4	0.2	7.4
	24	11.1	0.0	0.2	0.0	7.4
	31	11.3	0.0	0.0	0.2	7.2
W85	2	11.0	5.1	6.9	2.0	5.9
	10	11.3	1.0	4.1	0.7	7.1
	17	11.9	0.0	0.4	0.4	9.6
	24	12.1	0.0	0.0	0.1	9.3
	31	13.1	0.0	0.0	0.1	8.8
W80	2	10.7	6.8	6.7	2.3	5.2
	10	11.2	0.6	4.6	1.4	8.3
	17	12.0	0.1	0.4	1.0	9.8
	24	12.0	0.0	0.1	0.3	9.6
	31	13.0	0.0	0.0	0.4	9.7

**Table 5:** The variations in cotton yield and yield components under different irrigation treatments. Note: different lowercase letters indicate significant differences within different treatments (*p* < 0.05).

Treatments	Number of Bolls (×10^4^·hm^−2^)	Single Boll Weight (g)	Lint Percentage (%)	Cotton Yield (kg·hm^−2^)
W100	143.03 a	4.27 c	46.27 c	6111 a
W90	132.98 a	4.66 a	47.03 abc	6196 a
W85	134.33 a	4.07 d	47.97 ab	5469 b
W80	108.25 b	4.05 b	46.63 bc	4875 c

**Table 6:** Correlation between the measured indicators during the flowering and boll setting stage of cotton and cotton yield.

Indicators Types	Indicators	Yield-Related Analysis
Growth and Development	LAI	−0.997 **
	Stem diameter	0.688
	Plant height	0.568
Boll Formation Characteristics	Ringing rate	−0.946
	Shedding rate	0.879
Biomass	Aboveground biomass	−0.742
	Flower, bud and boll density	−0.369
Stress Resistance	MDA	−0.956 *
	POD	−0.952 *
	SOD	0.921
	SP	0.220
Yield Components	Number of bolls	0.857
	Single boll weight	0.137
	Lint percentage	−0.153

Note: * and ** mean correlation is significant at 0.05 and 0.01 level, respectively.

**Table 7:** The further distribution of irrigation frequency and water usage.

Treatments	Irrigation Number	Growth Periods	Dates	Irrigation Volume (m^3^/ha)
Control group (W90)	Five times in the flowering and boll setting stage of cotton	Sprouting	2023/4/23	450
		Seedling	2023/6/9	468
		Bud emergence	2023/6/19	334
			2023/6/29	334
			2023/7/9	401
			2023/7/14	334
		Flowering and boll setting	2023/7/21	535
			2023/7/28	468
			2023/8/4	468
			2023/8/11	401
			2023/8/18	401
		Boll opening	2023/8/25	267
Experimental group	Four times in the flowering and boll setting stage of cotton	Sprouting	2023/4/23	450
		Seedling	2023/6/9	468
		Bud emergence	2023/6/19	334
			2023/6/29	334
			2023/7/9	401
			2023/7/14	334
		Flowering and boll setting	2023/7/21	225/300//375/450/525/600/675
			2023/7/30	225/300//375/450/525/600/675
			2023/8/8	225/300//375/450/525/600/675
			2023/8/17	225/300//375/450/525/600/675
		Boll opening	2023/8/25	267
Experimental group	Three times in the flowering and boll setting stage of cotton	Sprouting	2023/4/23	450
		Seedling	2023/6/9	468
		Bud emergence	2023/6/19	334
			2023/6/29	334
			2023/7/9	401
			2023/7/14	334
		Flowering and boll setting	2023/7/21	600/750
			2023/8/2	600/750
			2023/8/14	600/750
		Boll opening	2023/8/25	267

## Data Availability

The data presented in this study are available in the graphs and tables provided in the manuscript.
